# Effect of meditation and prenatal education on fear and confidence in vaginal delivery

**DOI:** 10.3389/fpubh.2025.1712740

**Published:** 2025-12-10

**Authors:** Jun Zhu, Meiyun Zhou, Taotao Wang, Xiaoyan Ma, Dandan Yong

**Affiliations:** 1 Department of Obstetrics, Affiliated Hospital of Jiangsu University, Zhenjiang, Jiangsu, China; 2 Nursing Department of Staff Hospital, Affiliated Hospital of Jiangsu University, Zhenjiang, Jiangsu, China; 3 Department of Clinical Nutrition, Affiliated Hospital of Jiangsu University, Zhenjiang, Jiangsu, China

**Keywords:** meditation and relaxation, prenatal multimedia health education, vaginal delivery women, fear of childbirth, confidence in childbirth

## Abstract

**Objectives:**

To evaluate the synergistic effect of meditation and relaxation techniques combined with prenatal multimedia health education on reducing fear of childbirth, enhancing childbirth confidence, and improving postpartum outcomes in women undergoing vaginal delivery.

**Methods:**

A retrospective cohort study was conducted at a single tertiary hospital from February 2021 and February 2024. A total of 212 women who underwent vaginal delivery were included, with 102 in the treatment group receiving meditation, relaxation techniques, and multimedia health education alongside standard prenatal care, and 110 in the control group receiving only standard prenatal care. Clinical data collected included fear of childbirth scores (W-DEQ), confidence scores (CBSEI), anxiety levels (S-AI), depression scores (SDS, EPDS), and labor duration. Statistical analyses compared these measures between groups at different stages of pregnancy and postpartum.

**Results:**

Labor duration did not significantly differ between groups (*p* > 0.05). At 36 weeks of pregnancy, the treatment group had significantly lower W-DEQ scores (48.85 ± 6.22 vs. 59.35 ± 6.47; *p* < 0.001) and S-AI scores (51.64 ± 1.08 vs. 58.63 ± 1.04; *p* < 0.001), and higher CBSEI scores (7.88 ± 1.41 vs. 5.17 ± 1.62; *p* < 0.001). Postpartum, the treatment group showed higher GCQ (92.77 ± 1.65 vs. 86.53 ± 1.27; *p* < 0.001) and LAS scores (175.61 ± 16.53 vs. 117.34 ± 13.24; *p* < 0.001), along with lower EPDS scores (4.08 ± 1.22 vs. 5.26 ± 1.54; *p* < 0.001). No significant differences were observed in adverse reactions between groups (*p* > 0.05).

**Conclusion:**

Integrating meditation, relaxation techniques, and prenatal multimedia health education significantly reduces fear of childbirth, boosts childbirth confidence, and alleviates postpartum depression, without increasing adverse reactions. This approach is clinically beneficial for improving maternal mental health.

## 1 Introduction

Fear of Childbirth (FOC) is a significant psychological issue faced by many pregnant women, particularly those experiencing pregnancy for the first time (1). In recent years, with the widespread dissemination of modern medical information and a growing understanding of knowledge among pregnant women, many mothers have developed varying degrees of anxiety. This anxiety is often driven by fears of pain, unknown risks, and potential complications during labor. Such fear can negatively affect the childbirth process, leading to prolonged labor, increased pain, and even a higher rate of cesarean sections (2). In addition, a mother’s confidence in childbirth is closely linked to her previous experience, and a lack of confidence can exacerbate the fear of childbirth (3).

As a non-pharmacological intervention, meditation and relaxation training have received more and more attention in recent years in reducing maternal labor pain and relieving anxiety (4). Meditation and relaxation techniques reduces pain and fear during childbirth by adjusting the mother’s breathing rhythm, relaxing muscle tension, and improving psychological tolerance, thereby improving the self-efficacy of childbirth (5). Meditation and relaxation techniques have a significant positive impact on maternal control during delivery, helping mothers feel more confident and proactive throughout the delivery process. Additionally, prenatal health education plays a crucial role in enhancing pregnant women’s understanding of the childbirth process. Traditional prenatal education typically relies on verbal explanations or written materials provided by medical staff. However, with advancements in science and technology, multimedia tools have become increasingly integrated into health education. Through multimedia forms such as videos, animations, pictures and texts, pregnant women can gain a more intuitive and comprehensive understanding of the childbirth process and related information. This approach not only enhances their sense of control over labor but also effectively reduces their fear of the unknown (6). Additionally, prenatal multimedia education can cater to the individual needs of different pregnant women, fostering a greater sense of participation and empowerment (7). Therefore, it is hypothesized that combining meditation, relaxation techniques, and pain relief with prenatal multimedia health education can provide a dual physiological and psychological intervention. Meditation, relaxation, and pain relief help mothers relax and alleviate pain, while prenatal multimedia education strengthens their understanding and control over the childbirth process, thereby boosting overall confidence in delivery. This combined intervention model not only aligns with the needs of modern maternity care but also effectively reduces maternal fear of childbirth, ultimately optimizing the delivery experience.

Based on this, the aim of this study is to investigate the effect of combining meditation and relaxation pain relief with prenatal multimedia health education on the fear of childbirth and confidence in women undergoing vaginal delivery. Through scientifically designed interventions, this study seeks to provide effective nursing strategies for clinical practice, ultimately improving the childbirth experience for women, reducing fear of labor, enhancing confidence in childbirth, and promoting natural birth rates. Additionally, it aims to improve the health outcomes of both mothers and babies, offering a theoretical foundation and practical guidance for the promotion of non-pharmacological pain relief methods.

## 2 Materials and methods

### 2.1 Research flow chart

**Figure 1:**
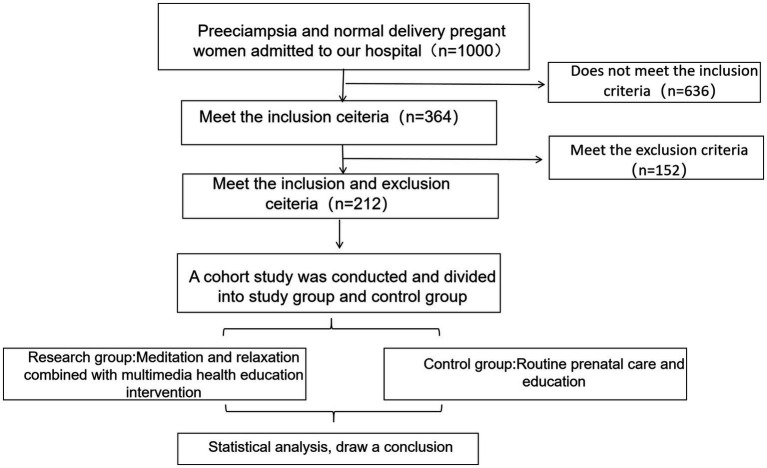
Research flow chart. No attrition occurred (retention 100%; loss to follow-up 0%). Outcome data were complete for all 212 participants.

Figure 1 shows the flow chart of this research.

### 2.2 Clinical data

A retrospective study was conducted on 1,135 pregnant women who received medical treatment and gave birth at our hospital between February 2021 and February 2024. According to the inclusion criteria and the mode of delivery (excluding cesarean sections), 212 pregnant women were included in the study cohort. This cohort consisted of the study group (*n* = 102), which received meditation relaxation combined with multimedia health education, and the control group (*n* = 110), which received conventional prenatal care and education. There were no statistically significant differences in the general characteristics between the two groups, as shown in Table 1. The current study was approved by the Ethics Committee of our university (approval number HJU202502059). Due to the retrospective design and anonymized data, written informed consent was waived by the Ethics Committee.

**Table 1:** Baseline characteristics comparison (*x_* ± s).

Characteristics	Research group (*n* = 102)	Control group (*n* = 110)	*χ^2^/*t-value	*P*-value
Age (years, *x_* ± s)	29.06 ± 5.72	28.94 ± 5.81	0.151	0.880
BMI (kg/m^2^, *x_* ± s)	27.14 ± 3.04	26.99 ± 3.28	0.345	0.731
Pregnancy times (times, *x_* ± s)	1.72 ± 0.61	1.68 ± 0.72	0.435	0.664
Gestational weeks (weeks, *x_* ± s)	20.17 ± 0.65	20.36 ± 0.77	1.934	0.055
Degree of education (*n*/%)			0.259	0.878
Junior high school and below	32 (31.37)	35 (31.82)		
High school and technical secondary school	41 (40.20)	47 (42.73)		
College degree and above	29 (28.43)	28 (25.45)		
Previous birth history (*n*/%)			0.000	0.992
Primipara	52 (50.98)	56 (50.91)		
Multiparous women	50 (49.02)	54 (49.09)		
Comorbidities (*n*/%)				
Gestational diabetes	8 (7.84)	7 (6.36)	0.302	0.583
Hypertension during pregnancy	11 (10.78)	9 (8.18)	0.420	0.517
Anemia	3 (2.94)	6 (5.45)	0.822	0.364
History of cesarean section (*n*/%)			2.172	0.141
Yes	25 (24.51)	18 (16.36)		
No	77 (75.49)	92 (83.64)		
History of miscarriage (*n*/%)			0.394	0.530
Yes	28 (27.45)	34 (30.91)		
No	84 (82.35)	86 (78.18)		
Monthly household income (Yuan), *n*/%			1.368	0.242
≤5,000	41 (40.20)	53 (48.18)		
>5,000	61 (59.80)	57 (51.82)		

Inclusion criteria: (1) Aged 18–40 years; (2) Singleton pregnancy with vaginal delivery; (3) Received prenatal meditation, relaxation training, or multimedia health education from 20 weeks of pregnancy until delivery; (4) Assessed by a physician as eligible for participation in this study; and (5) Able to understand and willing to participate, providing informed consent form during the study period.

Exclusion criteria: (1) Severe complications during pregnancy or delivery (e.g., postpartum hemorrhage, emergency cesarean section conversion); (2) Incomplete medical record or missing key data; (3) Multiple pregnancies; (4) Conditions such as placenta previa, placental abruption, cervical insufficiency, and patients with mental illness; (5) Conversion to cesarean section due to complications or other reasons during pregnancy; and (6) Receipt other interventions or treatments during pregnancy that could interfere with the study outcomes. Outcome data were ascertained from medical records and routine postpartum visits; no participant was lost to follow-up.

### 2.3 Treatment

The control group received routine delivery care. Before delivery, nursing staff provided parturients with an introduction to the delivery process, psychological counseling, and instruction on effective breathing and exertion techniques. During the first stage of labor, chest breathing was used, with breathing gradually accelerating from slow to fast during the active phase. Once cervical dilation reached ≥3 cm, parturients were transferred to the delivery room to prepare for delivery. In the second stage of labor, breath-holding and forceful pushing were utilized. During contractions, parturients were encouraged to perform relaxation exercises as needed to maintain physical strength and facilitate a smoother delivery.

The study group used mindfulness meditation and relaxation pain relief combined with phased posture management, and the prenatal care was the same as the control group.

1. Mindfulness meditation and relaxation pain relief. When the parturient assumed a specific posture, the nurse provided items such as balls or fruits to stimulate her imagination. For instance, if the parturient was holding an apple, the nurse would instruct her to focus on the apple and observe its texture, shape, and color. Subsequently, the nurse would guide her to close her eyes and patiently lead her through a relaxation exercise, encouraging her to imagine her body merging with the apple, exploring its color, appearance, and sensations. The parturient was then instructed to visualize everything she had imagined, take five deep breaths, slowly count to five, and then open her eyes.
2. Prenatal multimedia health education. The research group received regular prenatal multimedia health education sessions, which included a candid discussion of the disadvantages of cesarean delivery. Pregnant women were regularly organized into learn about perinatal knowledge. The skills and key aspects of vaginal delivery were explained using various formats, including videos, audio recordings, and printed materials. A simulated delivery scene was presented to help mothers understand the childbirth process accurately. Multimedia video materials were used to demonstrate proper breathing techniques, body positioning, and abdominal pressure application, guiding mothers in mastering these skills effectively. Additionally, the benefits of breastfeeding and appropriate breast care methods were explained. Pregnant women were informed about possible discomforts and precautions during delivery to ensure they were psychologically prepared. They were also encouraged to maintain a balanced diet and exercise appropriately to manage weight gain during late pregnancy. Exercises such as pregnancy gymnastics and yoga were taught to help correct pelvic tilt. WeChat and QQ groups were established to facilitate communication between nurses and patients, allowing for timely responses to mothers’ questions. The study group received a standardized program of mindfulness-based meditation and relaxation pain relief combined with prenatal multimedia health education.

Meditation and relaxation sessions were conducted once per week from 20 weeks of gestation until delivery, each lasting approximately 30–40 min, led by trained nurses certified in perinatal care. The training included guided breathing exercises, progressive muscle relaxation, and visualization (e.g., imagining tactile sensations while holding an object). Participants were encouraged to practice daily at home for 15 min using audio recordings provided by the team.

The multimedia prenatal health education component comprised eight structured sessions held biweekly from 20 to 36 weeks of gestation. Content was delivered through videos, illustrated slides, and short interactive quizzes, covering topics such as labor stages, pain management, and postpartum care. To ensure consistency and reproducibility, all sessions followed a standardized teaching manual and schedule, and adherence was monitored by supervising nurses. Both components were applied uniformly across participants in the intervention group.

### 2.4 Observation indicators

(1) Comparison of the duration of the first, second, third and total stages of labor between the two groups. (2) Fear of childbirth. The Wijma Delivery Expectancy/Experience Questionnaire (W-DEQ) (8) was used to assess the psychological state of pregnant women at 20 and 36 weeks of gestation.

The scale comprises 33 items, each scored from 0 to 5, resulting in a total score range of 0 to 165. Higher scores indicate greater severity of childbirth-related fear. Scores of 0–37 indicate no or mild fear, 38–65 indicate moderate fear, 66–84 indicate severe fear, and scores of 85 and above indicate extreme fear. (3) Confidence in childbirth. The Childbirth Self-Efficacy Inventory (CBSEI) (9) was used to assess the confidence of pregnant women in their ability to cope during labor, evaluated at 20 weeks and 36 weeks of gestation. The CBSEI is divided into two components: coping efficacy regarding expected labor pain and outcome efficacy regarding expected labor pain. Each component includes multiple items, with each item scored on a 10-point scale, ranging from 1 (no confidence) to 10 (completely confident). The scoring categories include: 1 point (no confidence), 2–3 points (very unconfident), 4–5 points (less confident), 6–7 points (somewhat confident), 8–9 points (very confident), and 10 points (completely confident). A higher total score indicates greater confidence in the childbirth process. (4) Postpartum depression score. The Edinburgh Postnatal Depression Scale (EPDS) (10) was used to assess maternal depression 1 week after delivery. The scale consists of 10 items, each scored from 0 to 3 points, resulting in a total score range of 0 to 30 points. Higher scores indicate a greater the risk of postpartum depression, with scores of 10 or above generally suggesting the possibility of postpartum depression. (5) Psychological state: One hour after delivery, the State Anxiety Inventory (S-AI) (11) and the Self-Rating Anxiety Scale (SAS) (12) were used to retrospectively assess the mother’s psychological state during labor. The S-AI evaluates a patient’s immediate feelings or experiences at a specific moment and consists of 20 items, each scored from 1 to 4, with a total score ranging from 20 to 80. Mothers responded based on their own experiences during delivery, with higher scores indicating higher levels of anxiety. The SAS also contains 20 items; each scored from 1 to 4. The total raw score is multiplied by 1.25 to obtain the standard score. A standard score of ≥50 indicates the presence of anxiety, with 50 to 59 indicating mild anxiety, 60 to 69 indicating moderate anxiety, and ≥70 indicates severe anxiety. (6) Labor comfort and sense of control during labor: Kolcaba’s General Comfort Questionnaire (GCQ) (13) was used to assess maternal comfort during labor, 1 hour after delivery. The GCQ contains 28 items across four dimensions: physical, psychological, spiritual, socio-cultural, and environmental. Each item is scored from 1 to 4, with higher total scores indicating greater comfort. The Labor Agentry Scale (LAS) (14) was used to evaluate the maternal sense of control during labor. The LAS consists of 29 items, each scored from 1 to 7, resulting in a total score range of 29 to 203. Higher scores reflect a stronger sense of control during labor.

#### 2.4.1 Reliability and validity of instruments

We used established instruments with prior evidence of content and construct validity (validated Chinese-language versions where available). In our sample, internal consistency was assessed for multi-item scales using Cronbach’s *α* (and McDonald’s *ω* where appropriate); values and 95% CIs are provided in Supplementary Table S1. All scales met conventional thresholds for acceptable reliability (≥0.80). Convergent and known-groups checks (prespecified) supported construct validity (see Supplementary Table S2). Instruments were administered and scored according to developers’ manuals, and scoring directions follow the descriptions above.

### 2.5 Statistical analysis

SPSS 24.0 statistical software was used. Before statistical analysis, the measurement data were tested for normal distribution and variance homogeneity to meet the requirements of normal distribution or approximate normal distribution, expressed as (*x_* ± s). The two groups were compared using the independent sample *t* test, with *n* (%) representing the count data and the χ^2^ test, with *p* < 0.05 indicating a statistically significant difference. For consistency, *p*-values are reported as uppercase “P,” using threshold notation for very small values (*p* < 0.001) and exact values to two decimals otherwise; the statistical test used for each comparison is specified in the Results and footnotes.

### 2.6 Sample size and power

We conducted a *post hoc* precision/power appraisal for two-sample comparisons (two-sided *α* = 0.05). With *n* = 102 (intervention) and *n* = 110 (control), the study has ~80% power to detect a standardized mean difference of approximately *d* = 0.39, ~90% power for *d* = 0.45, and ~95% power for *d* = 0.50. Observed effects on key psychological outcomes were larger than this benchmark, indicating adequate power for the study’s primary aims.

## 3 Results

### 3.1 Baseline data

The mean age of patients in the study group was 29.06 ± 5.72 years, while that in the control group was 28.94 ± 5.81 years, with no significant difference between the two groups (*t* = 0.151, *p* = 0.880). The mean BMI was 27.14 ± 3.04 kg/m^2^ in the study group and 26.99 ± 3.28 kg/m^2^ in the control group, with no significant difference between the groups (*t* = 0.345, *p* = 0.731). The average number of pregnancies was 1.72 ± 0.61 in the study group and 1.68 ± 0.72 in the control group, without any significant difference between the groups (*t* = 0.435, *p* = 0.664). The mean gestational age was 20.17 ± 0.65 weeks in the study group and 20.36 ± 0.77 weeks in the control group, also showing no significant difference between the groups (*t* = 1.934, *p* = 0.055). Additionally, there were no significant differences between the groups in education level, previous delivery history, comorbidities, history of cesarean sections, abortion history, or family monthly income (*p* > 0.05). Therefore, the baseline characteristics were comparable between the two groups, as shown in Table 1. A total of 212 participants were enrolled in this study (102 in the research group and 110 in the control group). All participants completed the final follow-up and contributed outcome data (retention 100%; loss to follow-up 0%). Because this was a retrospective, records-based cohort, a traditional response rate is not applicable; outcome data completeness was 100%.

### 3.2 Comparison of delivery time between the two groups

**Figure 2:**
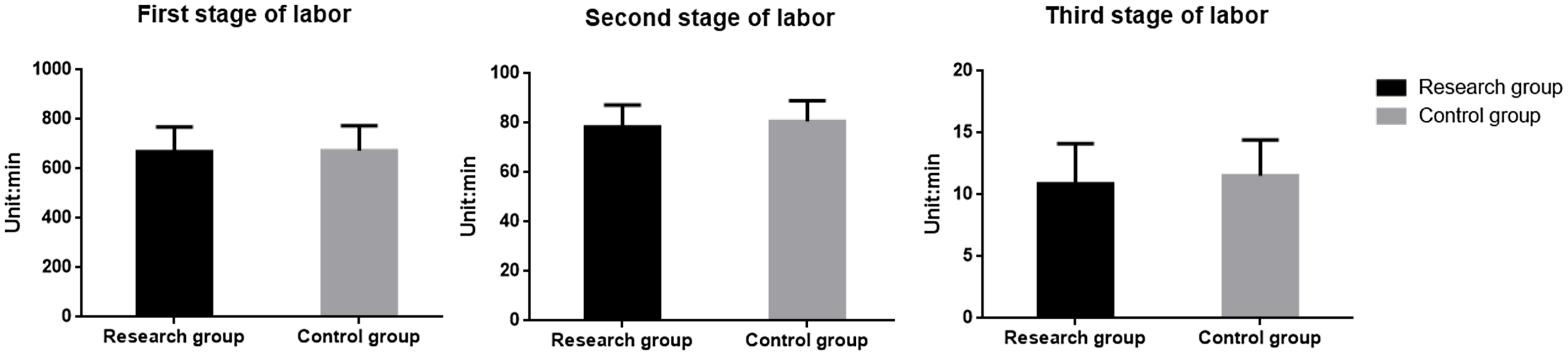
Comparison of the first, second and third stages of labor between the two groups.

In the observation group, the duration of the first stage [(668.54 ± 100.28) min vs. (673.29 ± 100.41) min], second stage [(78.41 ± 8.92) min vs. (80.63 ± 8.41) min], and third stage [(10.87 ± 3.26) min vs. (10.87 ± 3.26) min] of labor was shorter compared to the control group. However, these differences were not statistically significant (*t* = 0.344, 1.865, 1.586; *p* = 0.731, *p* = 0.064, *p* = 0.114), as shown in Figure 2.

### 3.3 Comparison of childbirth fear and childbirth confidence scores between the two groups at 20 weeks and 36 weeks of pregnancy

**Figure 3:**
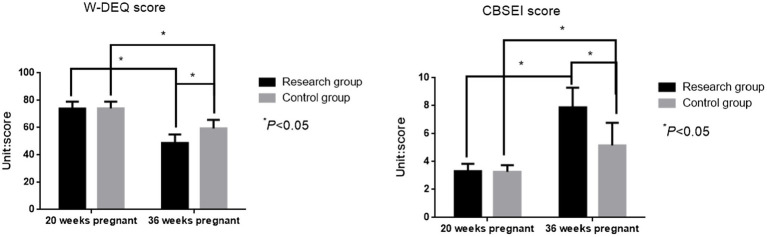
Comparison of W-DEQ scores and CBSEI scores at 20 weeks and 36 weeks of gestation between the two groups.

At 20 weeks of gestation, there was no significant difference between the two groups in the W-DEQ score [(74.01 ± 5.14) vs. (73.92 ± 5.26)] or the CBSEI score [(3.26 ± 0.48) vs. (3.31 ± 0.54)] (*t* = 0.126, 0.714; *p* = 0.900, *p* = 0.476). However, at 36 weeks of gestation, the W-DEQ score in the study group was significantly lower than that in the control group [(48.85 ± 6.22) vs. (59.35 ± 6.47)], while the CBSEI score was significantly higher in the study group [(7.88 ± 1.41) points vs. (5.17 ± 1.62) points] (*t* = 12.028, 12.948; *p* < 0.001, *p* < 0.001, Figure 3).

### 3.4 Comparison of psychological status between the two groups

**Figure 4:**
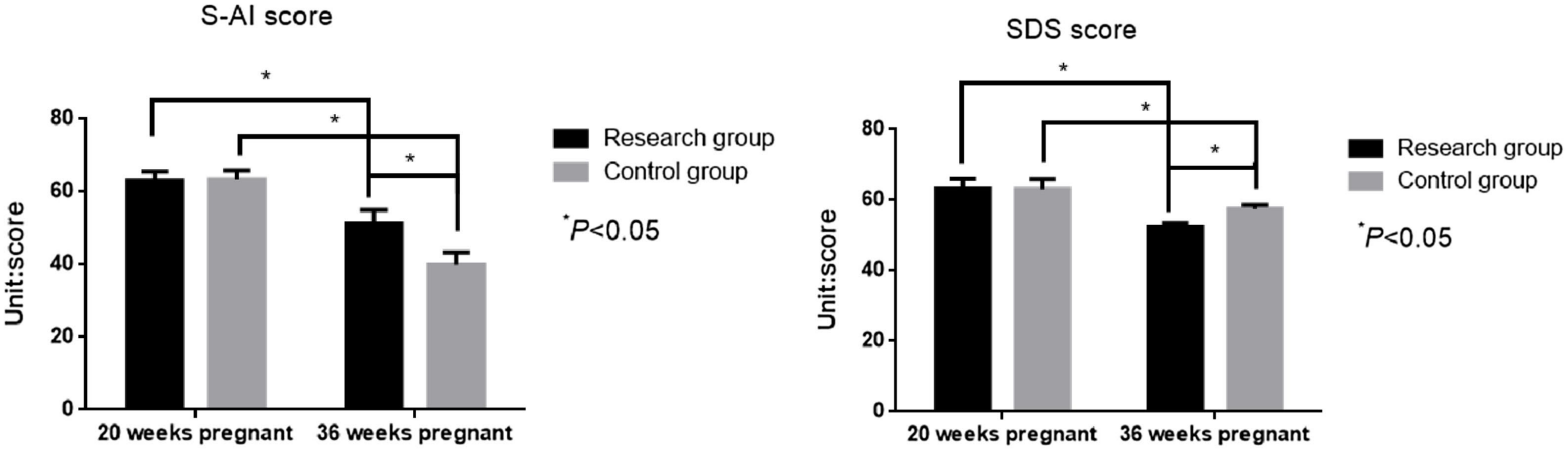
Comparison of S-AI scores and SDS scores before and after delivery between the two groups.

There was no significant difference in S-AI score [(61.41 ± 1.62) score vs. (61.34 ± 1.73) score] and SDS score [(63.32 ± 2.88) score vs. (63.27 ± 2.71) score] between the two groups at 20 weeks of pregnancy (*t* = 0.304, 0.130; *p* = 0.761, *p* = 0.897). The S-AI score [(51.64 ± 1.08) points vs. (58.63 ± 1.04) points] and SDS score [(57.76 ± 1.12) points vs. (52.34 ± 1.09) points] of the study group were lower than those of the control group at 36 weeks of pregnancy (*t* = 47.999, 35.662; *p* < 0.001, *p* < 0.001, Figure 4).

### 3.5 Comparison of labor comfort and sense of labor control between the two groups

**Figure 5:**
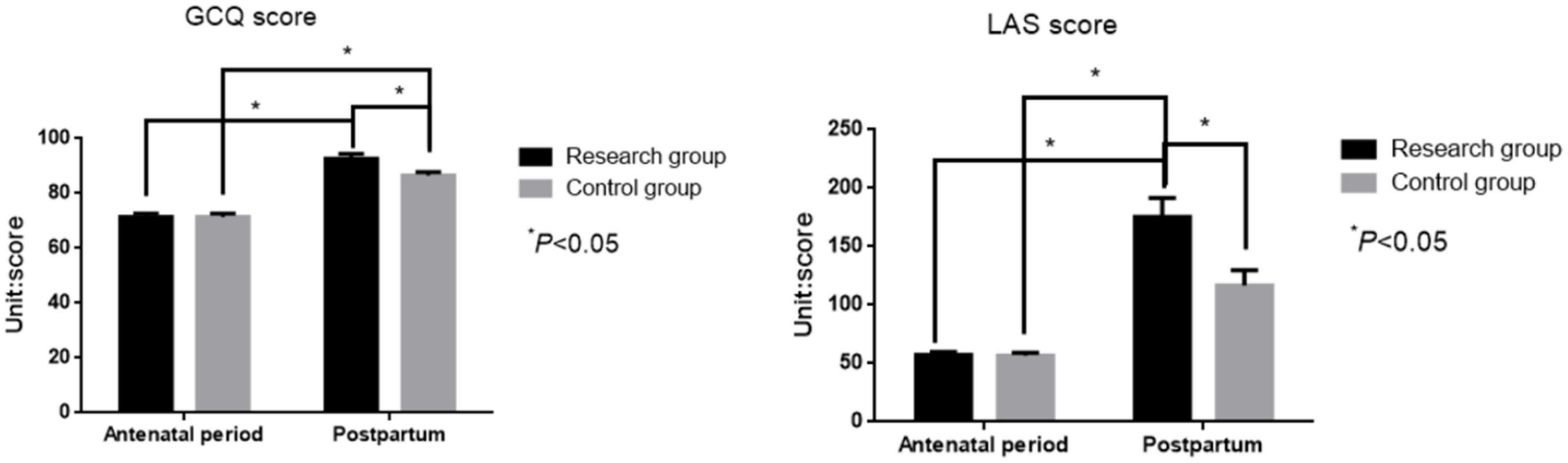
Comparison of GCQ scores and LAS scores before and after delivery between the two groups.

There was no significant difference in prenatal GCQ score [(71.41 ± 1.31) vs. (71.38 ± 1.53)] and LAS score [(56.48 ± 2.71) vs. (56.51 ± 2.83)] between the two groups (*t* = 0.079, 0.154; *p* = 0.937, *p* = 0.878, Figure 5). The postpartum GCQ score [(92.77 ± 1.65) points vs. (86.53 ± 1.27) points] and LAS score [(175.61 ± 16.53) points vs. (117.34 ± 13.24) points] of the study group were higher than those of the control group (*t* = 28.425, 30.984; *p* < 0.001, *p* < 0.001).

### 3.6 Comparison of postpartum depression scores between the two groups of pregnant women at 1 week after delivery

The EPDS scores [(4.08 ± 1.22) vs. (5.26 ± 1.54) points] of the study group were significantly lower than those of the control group, and the difference was statistically significant (*t* = 6.152, *p* < 0.001).

## 4 Discussion

In recent years, the clinical cesarean section rate has increased, driven by a range of complex factors. One key factor is that many parturients fear labor pain and lack adequate knowledge, leading to subjective requests for cesarean sections even in the absence of medical indications. While cesarean sections can eliminate labor pain, they are associated with severe postoperative pain that can hinder early maternal activity. This may increase the risk of complications, such as infections and deep vein thrombosis, and can negatively impact mother-infant bonding, delay the onset of lactation, and even lead to breastfeeding failure. The use of anesthetic drugs during surgery further elevates the risk of complications, potentially affecting both maternal and neonatal safety. Over time, the long-term risks of cesarean sections have become increasingly evident, including an elevated risk of complications such as ectopic pregnancy and placenta accreta in future pregnancies. Therefore, strict regulation of cesarean section rates to avoid unnecessary procedures and promote natural delivery has become a priority in obstetric clinical practice.

Natural vaginal delivery is a normal physiological process. Whether it can be delivered smoothly and naturally depends on many factors such as birth canal conditions, fetal size, productivity, and mental state. Factors such as maternal physical strength and mental state have a direct impact on fertility (15). Therefore, in addition to weight control and prevention of macrosomia, prenatal health education should also focus on maternal physical exercise and the relief of negative emotions. In addition, relevant studies point out that providing mothers with reasonable and effective nursing measures during delivery can ensure smooth vaginal delivery and reduce the incidence of complications (16, 17). Mindfulness meditation, relaxation and pain reduction techniques are relatively new forms of childbirth intervention. These methods aim to divert the mother’s attention, relax her nervous system, and consequently alleviate labor pain. This study showed that after the intervention of meditation, relaxation and pain reduction combined with multimedia health education, the maternal fear of childbirth in the study group was significantly reduced, and the W-DEQ score was significantly lower than that of the control group. These results are consistent with prior work indicating that meditation-based training can improve attentional control and emotion regulation and thereby reduce fear and anxiety during the peripartum period (18, 19). They also align with clinical guidance emphasizing that structured preparation and supportive care improve labor experiences and reduce unnecessary interventions (20). Alleviating the fear of childbirth not only reduces the psychological burden on mothers but also promotes smoother deliveries and minimizes unnecessary interventions, such as reducing the rate of cesarean sections. Therefore, addressing the fear of childbirth is crucial for enhancing maternal delivery experience and improving delivery outcomes. First, meditation and relaxation pain relief is a non-drug intervention method that helps mothers cope with pain and anxiety during childbirth by regulating breathing rhythm, relaxing muscle tension, and improving psychological tolerance. Studies have shown that meditation and relaxation training can guide mothers to shift their attention from pain and anxiety to breathing rhythm and imagined situations through mindfulness meditation, thereby relieving physical tension and reducing pain (21, 22). Mindfulness meditation is a technique that emphasizes focusing on the present moment. It helps mothers concentrate on their breathing, adjust body positions, and connect with their inner sensations, thereby reducing the perception of pain and enhancing their sense of self-efficacy during childbirth. Additionally, prenatal multimedia health education provides mothers with comprehensive information about the childbirth process, helping them to be psychologically prepared. Unlike traditional health education, which relies primarily on oral explanations or written materials, modern multimedia health education employs videos, animations, graphics, and text to offer a more intuitive understanding of each stage of childbirth and how to manage potential challenges. This multi-sensory approach enables mothers to gain a deeper understanding of the childbirth process, thereby enhancing their sense of control and confidence.

The magnitude of improvements in fear of childbirth and self-efficacy exceeded a small-to-moderate effect benchmark (*d* = 0.40), supporting clinical relevance. Findings align with prior reports that mindfulness-based relaxation and structured prenatal education enhance maternal coping and perceived control. Principal limitations include single-center, retrospective design and potential unmeasured confounding; nevertheless, internal consistency of measures was high and validity checks were supportive. Prospective multicenter trials are warranted.

Mechanisms of benefit. Our findings are biologically and psychologically plausible. Mindfulness-based meditation may reduce fear and anxiety by enhancing attentional control and cognitive reappraisal, dampening hypervigilance to pain/threat cues, and improving tolerance of labor sensations; these processes can increase childbirth self-efficacy and perceived control (14, 19). In parallel, structured prenatal education likely reduces uncertainty, sets accurate expectations, and provides skills (breathing/posturing/partner support), which together lower anticipatory fear and improve coping during labor (20). The joint effect of “skills + state training” may explain the concurrent improvements we observed across W-DEQ (fear), CBSEI (self-efficacy), S-AI/SDS/EPDS (anxiety/mood), and GCQ/LAS (comfort/agency).

In our cohort, higher childbirth self-efficacy (CBSEI) and greater perceived control (LAS) in the intervention group parallel prior observations that preparation and agency are linked to more favorable psychological outcomes around delivery (14, 20). Where studies have reported smaller or null effects, differences have often involved shorter or less intensive interventions, later initiation in pregnancy, or samples with lower baseline psychosocial risk factors that may attenuate measurable change.

Childbirth confidence is a mother’s confidence in her ability to cope with labor pain and various uncertain situations during childbirth (23). The level of childbirth confidence directly affects the mother’s sense of control over the childbirth process and is closely related to her childbirth experience. The results of this study showed that the maternal confidence in childbirth in the study group was significantly higher than that in the control group, indicating that meditation relaxation and pain reduction method combined with multimedia health education can effectively enhance maternal confidence in childbirth. This is similar to reports that targeted education and skills practice can increase agency and reduce fear, although effect sizes vary by setting and parity distribution (20, 24). Improved childbirth confidence not only enables mothers to cope more effectively with labor pain and other challenges but also reduces resistance to childbirth and increases the likelihood of natural birth. More importantly, enhanced childbirth confidence is closely linked to maternal and child health, as it helps mothers maintain a positive psychological state, promoting better postpartum recovery and fostering mother-infant bonding. Research also indicates that combining meditation, relaxation, and pain relief with multimedia health education can significantly improve the psychological well-being of pregnant women. The S-AI and SDS scores of pregnant women in the study group were significantly lower than those in the control group, indicating that the intervention effectively reduced maternal anxiety and depression during delivery. This pattern is directionally consistent with evidence that mindfulness-oriented and educational interventions reduce state anxiety and mood symptoms during pregnancy and postpartum (19, 25). These findings further support the potential of non-pharmacological psychological interventions to promote maternal mental health. Maintaining a positive psychological state not only facilitates a smoother delivery but also helps reduce postpartum psychological issues, such as postpartum depression. Postpartum depression is a common condition experienced by many mothers after childbirth, significantly affecting both maternal and infant health. This study demonstrated that EPDS scores in the study group were significantly lower than those in the control group 1 week after delivery, suggesting that this intervention effectively prevents and alleviate postpartum depression. Differences in absolute EPDS change across studies may reflect baseline severity, timing of assessment, and postnatal support models, which can moderate intervention impact.

Comparison with existing literature and clinical relevance. Our results align with recent trials showing that mindfulness-oriented interventions reduce fear/anxiety and improve birth experience (14, 19), and with syntheses indicating that structured prenatal education improves fear, pain experience, and postpartum mood (20). Extending prior work, our cohort demonstrates concurrent gains across multiple validated domains (fear, self-efficacy, anxiety/mood, comfort, perceived control) within a single real-world program, which strengthens the case for combined meditation+education models in routine care. Where studies report smaller or null effects, heterogeneity in intervention dose/timing and baseline psychosocial risk likely attenuates impact; in contrast, our standardized schedule and early start (20 weeks) may have amplified benefits (20). Clinically, improved self-efficacy and perceived control are relevant because lower labor agentry has been linked to adverse downstream mental health outcomes (14), supporting the potential of these interventions to confer benefits beyond delivery. Taken together, the consistency with contemporary evidence, the breadth of outcomes, and complete follow-up underscore the program’s feasibility and translational relevance for prenatal services.

The methodological framework and statistical analyses applied in this study are robust, and the conclusions are directly supported by the observed results. We have improved clarity and consistency in reporting test statistics and *p*-values, and we prespecified reliability/validity checks, which strengthens the reliability and reproducibility of the findings. Together with the use of validated instruments and complete follow-up data, these improvements further support the internal validity of the study and confidence in its conclusions.

This study was conducted at a single tertiary hospital and used a retrospective cohort design, which may limit causal inference and external validity. Although we included all eligible women during the study window, the sample size (*n* = 212) is modest, and unmeasured confounding or selection bias cannot be fully excluded. While we have now detailed the intervention’s content, schedule, and standardization in Methods 2.3, the protocol reflects a single-center implementation and may require adaptation in other settings. Generalizability may therefore be limited to similar patient populations and service models. Future work should employ multicenter, prospective (ideally randomized) designs with larger and more diverse samples, prespecified intervention “dose” and fidelity checks, and longer postpartum follow-up to confirm durability of effects and optimize personalization (e.g., tailoring by baseline fear, parity, or psychosocial risk).

Strengths and contributions. Compared with prior studies, our work offers several strengths: (i) a clearly defined routine-care control group; (ii) comprehensive use of validated instruments spanning fear (W-DEQ), self-efficacy (CBSEI), anxiety/depressive symptoms (S-AI/SAS/EPDS), and comfort/agency (GCQ/LAS) (18, 20); (iii) complete outcome ascertainment with 100% retention and no loss to follow-up; and (iv) a real-world, multi-year hospital cohort that enhances external validity. Together with concordant findings from meditation- and education-based studies (18, 20) and the recognized importance of perceived agency around childbirth (14), our results support the clinical utility of combining meditation/relaxation with multimedia health education in routine prenatal care. The study uses validated instruments across multiple domains, demonstrates robust intervention effects, and advances a non-pharmacological approach well-suited to contemporary obstetric practice.

In summary, the combination of meditation, relaxation, and pain relief techniques with prenatal multimedia health education significantly reduces fear of childbirth, enhances childbirth confidence, and alleviates symptoms of postpartum depression in women undergoing vaginal delivery. This non-pharmacological intervention not only aligns with the needs of modern obstetric care but also improves the maternal childbirth experience and has substantial clinical applicability and promotional value. However, this study has several limitations. First, it was a single-center study with a relatively small sample size, which may limit the generalizability of the results. Future research should consider multi-center studies with larger sample sizes to further validate the effectiveness of this intervention. Second, the intervention methods used in this study were relatively fixed. Future research could explore more personalized intervention programs to address the specific needs of different mothers. Additionally, further research could investigate the applicability of this intervention in diverse maternal groups, such as women with high-risk pregnancies or multiparous women. Finally, prospective randomized studies with standardized intervention dosing and longer postpartum follow-up would help delineate durability of effects and clarify which components (meditation vs. multimedia education) contribute most to benefit.

## Data Availability

The raw data supporting the conclusions of this article will be made available by the authors, without undue reservation.
