# Supplementary material for: Effect of meditation and prenatal education on fear and confidence in vaginal delivery
Source: Front Public Health. 2025 Dec 10;13:1712740. doi: 10.3389/fpubh.2025.1712740 (PMC12728045; doi:10.3389/fpubh.2025.1712740)
Supplement: Supplementary file 1 [file Table_1.DOCX]

**Supplementary Tables**

**Table S1. Reliability of instruments published benchmarks**

| Instrument | Items (k) | Sample size (n) | Cronbach’s α (benchmark) | Source (first author, year) | Notes |
| --- | --- | --- | --- | --- | --- |
| W-DEQ | 33 | — | 0.84 | Validation study | W-DEQ-A validation; α=0.84 |
| CBSEI (total/short) | 32 (short) / 62 (orig.) | — | 0.95 | Validation study | Very high internal consistency |
| S-AI (STAI-State) | 20 | — | 0.91 | Translated version | Acceptable to excellent |
| SAS | 20 | — | 0.82 | Validation study | Acceptable internal consistency |
| GCQ | 28 | — | 0.81 | Validation study | Adult validation sample |
| LAS | 29 | — | 0.91–0.98 | Validation study | Often ≥0.90 across studies |
| EPDS | 10 | — | 0.79 | Validation study | Good construct validity |

Note: Values shown are published reliability benchmarks for validated versions of each instrument. They are provided to document expected measurement performance and are not estimates from the current cohort.

**Table S2. Validity evidence constructs and known-groups**

*Part A. Hypothesis-driven correlations (construct validity)*

| Pairs (expected direction) | Expected direction | Evidence basis | This cohort result |
| --- | --- | --- | --- |
| W-DEQ vs S-AI (+) | Positive | Anxiety increases with fear (literature) | Not assessed (retrospective aggregate-only) |
| W-DEQ vs SAS (+) | Positive | Anxiety increases with fear (literature) | Not assessed (retrospective aggregate-only) |
| W-DEQ vs EPDS (+) | Positive | Depressive symptoms rise with fear (literature) | Not assessed (retrospective aggregate-only) |
| W-DEQ vs CBSEI (–) | Negative | Higher efficacy reduces fear (literature) | Not assessed (retrospective aggregate-only) |
| W-DEQ vs GCQ (–) | Negative | Comfort inversely related to fear (literature) | Not assessed (retrospective aggregate-only) |
| W-DEQ vs LAS (–) | Negative | Perceived control inversely related to fear (literature) | Not assessed (retrospective aggregate-only) |

*Part B. Known-groups validity by parity*

| Outcome | Known-groups hypothesis | Evidence basis | This cohort result |
| --- | --- | --- | --- |
| W-DEQ (fear) | Primiparas > Multiparas | Commonly observed in validation studies | Not assessed (retrospective aggregate-only) |
| CBSEI (self-efficacy) | Primiparas < Multiparas | Commonly observed in validation studies | Not assessed (retrospective aggregate-only) |
